# Efficacy of Magnesium Sulfate in Addition to Melatonin Therapy in Neonates With Hypoxic-Ischemic Encephalopathy

**DOI:** 10.7759/cureus.21163

**Published:** 2022-01-12

**Authors:** Muhammad H Khan, Qurrat-ul Ann, Muhammad S Khan, Nadeem Ahmad, Moiz Ahmed

**Affiliations:** 1 Department of Neonatology, Pakistan Institute of Medical Sciences, Islamabad, PAK; 2 Department of Neonatology, Pakistan Atomic Energy Commission General Hospital, Islamabad, PAK; 3 Department of Neonatology, Jinnah Postgraduate Medical Centre, Karachi, PAK

**Keywords:** neonates, mgso4, melatonin, magnesium sulfate, hie, hypoxic-ischemic encephalopathy

## Abstract

Background: One of the most important causes of neonatal deaths in developing nations is birth asphyxia. Though the probability of a complete recovery is very low, hypoxic-ischemic encephalopathy (HIE) associated with asphyxia can be managed with multiple treatment options. The study evaluated the efficacy of the addition of magnesium sulfate (MgSO_4_) to melatonin therapy in neonates with HIE.

Methodology: A prospective, observational study was conducted in the department of neonatal intensive care, Pakistan Institute of Medical Sciences Hospital, Islamabad, Pakistan from October 2020 to March 2021. All neonates with an Appearance, Pulse, Grimace, Activity, and Respiration (APGAR) score of less than five at five minutes, umbilical blood pH of less than 7.0, and moderate neonatal encephalopathy as detected on the modified Sarnat score which is a clinical tool used for the assessment of the severity of HIE were included in the study. Neonates with congenital abnormalities, intrauterine growth retardation, neonatal sepsis, and infants born to mothers with diabetes mellitus type 2 were excluded from the study. The infants were randomly assigned to either of the groups, i.e., i) group 1 included neonates who were administered at least three doses of magnesium sulfate (MgSO_4_) infusion in addition to melatonin, or ii) group 2 included neonates who were administered melatonin only. Blood samples of all neonates were evaluated and compared between the two groups.

Results: A total of 90 neonates with HIE were included in the study. There was a predominance of female neonates. The mean ages of babies in group 1 and group 2 were 37.2 ± 0.43 and 37.3 ± 0.59 weeks, respectively. The mean weight on the term was 2.88 ± 0.11 and 2.89 ± 0.10, respectively. The Apgar score at 5 mins in group 1 was 1.73 ± 0.81 while in group 2, 1.82 ± 0.94. It was found that there was a more significant improvement in pH after 3 days and after one week of treatment in group 1 as compared to group 2. The mean pH in babies after three days of intervention was 7.23 ± 0.03 in group 1 which was significantly better than group 2 (p<0.0001). After seven days, the mean normalized to 7.39 ± 0.04 in group 1 (p < 0.0001). It was found that in patients in group 1, the mortality was lower than in group 2 (p < 0.0001).

Conclusion: HIE patients who were administered melatonin in combination with magnesium sulfate yielded better patient outcomes. Thus, it was concluded that the use of magnesium sulfate as dual therapy with melatonin improved patient outcomes for HIE. However, it is recommended that similar studies are conducted with a wider range of parameters, such as duration of hospital stay and assessment of the neurological outcomes of the patients.

## Introduction

The World Health Organization indicated that about 16,000 children under the age of five die per day. The most frequent causes of child mortality are infectious diseases, preterm, and intrapartum-associated complications [1]. In third world countries, a major cause of childhood mortality is hypoxic-ischemic encephalopathy (HIE), with the incidence of asphyxia ten times higher than that in more developed countries [2].

Melatonin, chemically N-acetyl-5-methoxytryptamine, is a hormone released by the pineal gland and is considered to have multiple functions in the human body. Melatonin plays an active role in the maintenance of physiological functions such as regulating the circadian rhythm, contributing to reproductive, cerebrovascular neuroendocrine, and neuroimmunological activities along with its effect as an anti-inflammatory and an antioxidant [3-5]. Through its receptor and non-receptor actions, melatonin plays a crucial role as an antiapoptotic and anti-excitotoxic, thereby serving as a potent neuroprotector. Melatonin reduces the formation of proinflammatory molecules and inhibits cyclooxygenase activity, and is thus considered as an immune modulator [5].

Recent studies have also revealed that melatonin serves as a regulator of the blood-brain barrier, nitric oxide synthase, and various other antioxidant enzymes. The anti-apoptotic activity of melatonin markedly reduces the formation of proapoptotic substances thus maintaining mitochondrial permeability [6]. The analgesic effects of melatonin are also well known and are used in minor procedures to avoid procedural pain as it diminishes the release of proinflammatory cytokines in babies born preterm, who are suffering from respiratory distress, or infants with bacterial septicemia [7].

In the year 2001, Fulia et al. revealed that there was a significant reduction in levels of MDA and nitrates in the serum of asphyxiated newborn children, which was associated with melatonin administration in the first 6 hours after birth [8]. However, not enough clinical trials have been conducted with a large sample size to ascertain the effects and benefits of melatonin in the management of children with HIE. At present, hypothermia is considered to be the single most appropriate treatment for the management of HIE in neonates. Recently, many studies have been evaluating the association between hypothermia and melatonin in conjunction with magnesium sulfate (MgSO_4_) infusion use to enhance the neuroprotective effect of the brain and improve patient outcomes [9,10]. Thus, the present study aimed at evaluating the effectiveness of melatonin administration in addition to MgSO_4_ in improving patient outcomes.

## Materials and methods

A prospective, observational study was conducted in the department of neonatal intensive care, Pakistan Institute of Medical Sciences Hospital, Islamabad, Pakistan, from October 2020 to March 2021. The study was conducted after the ethical approval from the institutional review board was procured (Reference # IRB/P-54564/PIMS). Informed verbal and written consent was obtained from the parents of the neonates recruited in the study. A non-probability convenience sampling technique was employed for the recruitment of the participants. 

All neonates with an Appearance, Pulse, Grimace, Activity, and Respiration (APGAR) score of less than 5 at 5 minutes, umbilical blood pH of less than 7.0, and moderate neonatal encephalopathy as detected on the modified Sarnat score which is a clinical tool used for the assessment of the severity of HIE were included in the study. Neonates with congenital abnormalities, intrauterine growth retardation, neonatal sepsis, and infants born to mothers with diabetes mellitus type II were excluded from the study. 

A total of 90 neonates with HIE were included in the study. They were randomly assigned to either of the groups, i.e., i) group 1 included neonates who were administered at least three doses of magnesium sulfate (MgSO_4_) infusion (250 mg/kg per day intravenous over 60 minutes at days 0, 1, and 3 in addition to five doses of melatonin (10 mg/kg daily enteral for five consecutive days), or ii) group 2 included neonates who were administered the similar dose of melatonin only.

Blood samples were collected by a trained nurse from all neonates at presentation, on day 3, and on day 7. Complete blood count, serum electrolytes including serum magnesium concentrations, and arterial blood gases were assessed. Arterial blood pressure was regularly documented. All neonates were divided into groups according to the blood pressure categories. Normal arterial blood pressure in neonates was set as (40-65) mmHg whereas hypotension was considered at levels below 40 mmHg. Throughout the study, the patients were strictly monitored for any adverse outcomes or side effects such as respiratory depression or hypotension.

SPSS version 25 (IBM Corp., Armonk, NY) was used to run data analysis. Comparison between the two studied groups was done using the chi-square test and student t-tests to find out the associations between the dependent and the independent variables. A p-value equal to or less than 0.05 was considered statistically significant.

## Results

The baseline patient characteristics are provided in Table 1. The study showed a predominance of female neonates. The mean ages of babies in group 1 and group 2 were 37.2 ± 0.43 and 37.3 ± 0.59 weeks, respectively. The mean weight on the term was 2.88 ± 0.11 and 2.89 ± 0.10, respectively. The APGAR score at 5 min in group 1 was 1.73 ± 0.81 while in group 2, 1.82 ± 0.94 (Table 1).

**Table 1:** Comparison between the two studied groups according to demographic data and APGAR scores APGAR - Appearance, Pulse, Grimace, Activity, and Respiration

Parameter	Group 1	Group 2	P-value
Sex			0.421
Male	14 (31.1%)	18 (40%)	
Female	32 (71.1%)	27 (60%)	
Gestational age (wks)			0.982
Range	36-38	36-38	
Mean ± SD	37.2 ± 0.43	37.3 ± 0.59	
Median	37	37	
Weight (Kg)			0.979
Range	2.67-3.0	2.65-2.98	
Mean ± SD	2.88 ± 0.11	2.89 ± 0.10	
Median	2.95	2.96	
Apgar score at 5 min			0.861
Range	1.0-3.0	1.0-3.0	
Mean ± SD	1.73 ± 0.81	1.82 ± 0.94	
Median	1.6	1.4	
Apgar score at 10 min			0.61
Range	2.0-5.0	2.0-5.0	
Mean ± SD	3.41 ± 1.3	3.49 ± 1.2	
Median	3.5	3.66	

Table 2 compared the parameters after treatment with magnesium sulfate (MgSO_4_) in addition to melatonin (group 1) versus group 2 (melatonin only). It was found that there was a more significant improvement in pH after three days and after one week of treatment in group 1 as compared to group 2. The mean pH in babies after three days of intervention was 7.23 ± 0.03 in group 1, which was significantly better than group 2 (p < 0.0001). After seven days, the mean normalized to 7.39 ± 0.04 in group 1 (p < 0.0001) (Table 2).

**Table 2:** Comparison between the two studied groups according to pH MgSO_4_: magnesium sulfate

Parameter	Group 1	Group 2	P-value
Before MgSO_4_ infusion (at enrollment)			0.281
Range	7.03-7.14	7.05-7.17	
Mean ± SD	7.07 ± 0.02	7.08 ± 0.04	
Median	7.06	7.07	
After 3 days			<0.0001
Range	7.20-7.31	7.15-7.24	
Mean ± SD	7.23 ± 0.03	7.17 ± 0.05	
Median	7.22	7.18	
After 7 days			<0.0001
Range	7.32-7.44	7.16-7.28	
Mean ± SD	7.39 ± 0.04	7.2 ± 0.03	
Median	7.38	7.22	

Upon assessing the magnesium levels in each group, it was found that the difference was significant. At baseline, the mean magnesium levels were 1.71 ± 0.12 and 1.75 ± 0.09 in group 1 and group 2, respectively. However, in three days of infusion, the levels rose to 2.3 ± 0.10 and 1.99 ± 0.09, respectively (p < 0.0001). Similarly, by the seventh day of treatment, the magnesium levels in group 1 versus group 2 were 2.39 ± 0.10 and 2.14 ± 0.07, respectively (Table 3).

**Table 3:** Comparison between the two studied groups according to serum magnesium level in mg/L MgSO_4_: magnesium sulfate

Parameter	Group 1	Group 2	P-value
Before MgSO_4_ infusion (at enrollment)			0.299
Range	1.60-1.90	1.60-1.90	
Mean ± SD	1.71 ± 0.12	1.75 ± 0.09	
Median	1.79	1.77	
After 3 days			<0.0001
Range	2.10-2.40	1.85-2.05	
Mean ± SD	2.3 ± 0.10	1.99 ± 0.09	
Median	2.24	1.91	
After 7 days			<0.0001
Range	2.33-2.50	2.10-2.25	
Mean ± SD	2.39 ± 0.10	2.14 ± 0.07	
Median	2.41	2.15	

Table 4 shows the comparison of adverse effects in both groups. It was found that in patients in group 1, the mortality was lower than in group 2 (p < 0.0001) (Table 4).

**Table 4:** Comparison of distribution of adverse outcomes between the two groups

Parameter	Group 1 (n=45)	Group 2 (n=45)	P-value
Deaths	29 (64.4%)	39 (86.7%)	0.014
Seizure	28 (62.2%)	25 (55.6%)	0.52
Thrombocytopenia	34 (75.6%)	28 (62.2%)	0.172
Renal failure	20 (44.4%)	20 (44.4%)	0.832
Hypotension			0.483
Severe	19 (42.2%)	22 (48.9%)	
Mild to moderate	19 (42.2%)	17 (37.8%)	
Total	37 (82.2%)	39 (86.7%)	
Intracranial hemorrhage			0.153
< grade 3	43 (95.56%)	45 (100%)	
>= grade 3	2 (4.44%)	0 (0%)	
Total	45 (100%)	45 (100%)	

## Discussion

HIE among neonates causes damage to neurons and white matter, resulting in a significant rise in neonatal mortality [10]. Neurological injury in these cases occurs due to a lack of mitochondrial energy and a much higher expression of excitatory neurotransmitters in neonates [11].

The current study analyzed the efficacy of melatonin and magnesium sulfate (MgSO_4_) in the management of HIE among neonates. The results showed that both study groups had a lower pH which was indicative of acidosis. Low magnesium levels were also noted in both groups prior to the administration of MgSO_4_. However, after MgSO_4_ infusion, group 1 showed improved levels of magnesium on day two and day six while group 2 showed a very slight rise in magnesium levels. Improvements were more distinctively seen among patients of group 1, who were treated with MgSO_4_ in addition to melatonin.

A randomized controlled trial that evaluated the efficacy of melatonin in the management of infants with HIE revealed that patients suffered from fewer episodes of seizures and showed minimal signs of white matter injury on MRI, with much lower morbidity and mortality rates following treatment with melatonin [12]. These findings were consistent with our results, which also showed a slight improvement in patient condition following monotherapy with melatonin, even though much better results were seen in patients on dual therapy.

Our findings revealed that patients given melatonin in combination with MgSO_4_ yielded better patient outcomes. The role of MgSO_4_ in HIE was evaluated by Tagin et al., who reported that MgSO_4_ was associated with significant improvement in patient well-being and had no side effects [13]. Another study by Siddiqui et al. also reported similar results and revealed that MgSO_4_ played a role in improving neonatal reflexes and reducing the duration of seizures without any side effects [14].

The results of our study were inconsistent with those of Galinsky et al., who reported that MgSO_4_ led to no improvement in the neurological symptoms of the patient, with an absence of association between MgSO_4_ and improved outcomes among children at school-going age [15]. A similar study also revealed conflicting results and reported a lack of neurological impact of MgSO_4_ in cases of HIE [16]. This implies that though MgSO_4_ shows significant clinical results in neonates and infants, it does not improve outcomes of children belonging to the school-going age group. This indicates that the long-term benefits of MgSO_4_ may be minimal in improving neurodevelopmental outcomes [17].

The results of the current study were limited by the small sample size and the lack of follow-up by patients, which made it difficult to assess the neurodevelopmental outcomes of the babies belonging to both groups. Further studies are recommended to ascertain these findings in our population which is a resource-restrained region. 

## Conclusions

Our findings revealed that patients given melatonin in combination with magnesium sulfate yielded better patient outcomes. Thus, it was concluded in the study that the use of magnesium sulfate as dual therapy with melatonin improved patient outcomes for HIE. However, it is recommended that similar studies are conducted with a wider range of parameters, such as duration of hospital stay and assessment of the neurological outcomes of the patients.
